# Electrospun Nanofibers Encapsulated with Natural Products:  A Novel Strategy to Counteract Skin Aging

**DOI:** 10.3390/ijms25031908

**Published:** 2024-02-05

**Authors:** Diletta Serra, Giuseppe Garroni, Sara Cruciani, Donatella Coradduzza, Aleksei Pashchenko, Evzen Amler, Giorgio Pintore, Rosanna Satta, Maria Antonietta Montesu, Yvonne Kohl, Carlo Ventura, Margherita Maioli

**Affiliations:** 1 Department of Biomedical Sciences, University of Sassari, Viale San Pietro 43/B, 07100 Sassari, Italy; dilettaserra9@gmail.com (D.S.); giugarroni21@gmail.com (G.G.); sara.cruciani@outlook.com (S.C.); donatella.coradduzza0@gmail.com (D.C.); alexpko@seznam.cz (A.P.); 2 R&D Laboratory Center, InoCure s.r.o., Politických Veziu 935/13, 110 00 Prague, Czech Republic; 3 Department of Biophysics, Second Faculty of Medicine, Charles University, V Uvalu 84, 150 06 Prague, Czech Republic; 4 University Centre for Energy Efficient Buildings, Czech Technical University in Prague, Trinecka 1024, 273 43 Bustehrad, Czech Republic; amler@seznam.cz; 5 Department of Medicine, Surgery and Pharmacy, University of Sassari, 07100 Sassari, Italy; pintore@uniss.it; 6 Department of Medical, Surgical and Experimental Sciences, University of Sassari, 07100 Sassari, Italy; rsatta@uniss.it (R.S.); mmontesu@uniss.it (M.A.M.); 7 Fraunhofer Institute for Biomedical Engineering IBMT, Joseph-von-Fraunhofer-Weg 1, 66280 Sulzbach, Germany; yvonne.kohl@ibmt.fraunhofer.de; 8 Laboratory of Molecular Biology and Stem Cell Engineering, National Institute of Biostructures and Biosystems-Eldor Lab, Innovation Accelerator, CNR, Via Piero Gobetti 101, 40129 Bologna, Italy; ventura.vid@gmail.com; 9 Center for Developmental Biology and Reprogramming-CEDEBIOR, Department of Biomedical Sciences, University of Sassari, Viale San Pietro 43/B, 07100 Sassari, Italy

**Keywords:** stem cells, molecular mechanisms, nanofibers, skin aging, *Helichrysum italicum*, bioactive molecules, nanosystem, drug delivery

## Abstract

The skin is the primary tissue affected by wounds and aging, significantly impacting its protective function. Natural products are widely used in cosmetics, representing a new approach to preventing age-related damage. Nanomedicine combines nanotechnology and traditional treatments to create innovative drugs. The main targets of nanotechnological approaches are wound healing, regeneration, and rejuvenation of skin tissue. The skin barrier is not easily permeable, and the creation of modern nanodevices is a way to improve the passive penetration of substances. In this study, *Helichrysum italicum* oil (*HO*) was combined with different types of electrospun nanofibers to study their protective activity on the skin and to evaluate their future application for topical treatments. In the present research, we used biodegradable polymers, including polyvinyl alcohol (PVA) and polyvinylpyrrolidone (PVP), which were characterized by a scanning electron microscope (SEM). All results show a positive trend in cell proliferation and viability of human skin stem cells (SSCs) and BJ fibroblasts pre-treated with combined nanofibers and then exposed to UV stress. Gene expression analysis revealed the activation of a molecular rejuvenation program in SSCs treated with functionalized nanofibers before UV exposure. Understanding the mechanisms involved in skin changes during aging allows for the future application of nanomaterials combined with *HO* directly to the patients.

## 1. Introduction

The human skin is constantly exposed to internal and external stimuli, affecting its functionality. Wrinkles, skin dryness, reduced barrier integrity, and thinning of the epidermis appear with age [1]. Intrinsic and extrinsic factors influence skin aging. Intrinsic aging is the result of genetic factors and physical changes that appear during the normal aging process, while extrinsic aging is accelerated by environmental factors [2]. Extrinsic aging is synonymous with photoaging, as it occurs after exposure to UV radiation, inducing important consequences on the integrity of exposed skin [3,4]. Senescent cell numbers increase with age and contribute to age-related skin changes and pathologies. These types of cells are characterized by an inability to proliferate, resistance to apoptosis, and secretion of factors that promote inflammation and tissue deterioration [5,6,7]. Stem cell self-renewal and differentiation is regulated by the expression of the main stemness genes: the octamer-binding transcription factor 4 (Oct-4), the sex-determining region Y-box 2 (Sox-2), and the homeobox protein (NANOG). During aging, stem cells gradually lose their differentiation potential, while contemporary cells undergo a downregulation of stemness gene expression [8,9]. Premature senescence of stem cells could be one of the principal causes of skin aging, characterized by a progressive decline to replace damaged elements. Also, Bmi1, prevents premature senescence. It maintains the stem cell pool by downregulating genes involved in senescence and/or telomerase reverse transcriptase activity (TERT) [10]. TERT is involved in stabilizing telomeres, preventing their shortening after each cell division. Telomerase is highly expressed in proliferating cells while being drastically downregulated after differentiation and during cellular aging [9,10].

The skin provides an efficient barrier against external factors and regulates the flow of chemicals, thus counteracting the transition of hydrophilic and hydrophobic drugs. Optimizing local drug delivery represents an important challenge in cosmesis [11,12,13]. Skin treatments depend on the molecules to be delivered and the way of administration. Molecules can be delivered to implement rejuvenation and regeneration processes. In this sense, nanomedicine opened a new frontier for pharmacotherapeutic approaches that can improve the passive penetration of drugs [14,15]. Currently, nanoformulations are widely applied in cosmetic products [1,16]. Electrospun fibers are particularly intriguing for their versatility in the biomedical field [17]. Electrospun nanofibers present many advantages, including a large surface-to-volume ratio, low density, high porosity, and minimal pore size compared to conventional fibers. They have several applications, such as filtration membranes, cosmetics, drug delivery, biomedical scaffolds, and wound dressings [18,19,20]. Nanofibers have a small diameter of 1–100 nm and can be classified into three types: polymeric nanofibers, inorganic nanofibers, and organic/inorganic composite nanofibers. Due to their properties, polymeric nanofibers are used as carriers and employed in drug delivery [21].

Electrospinning is an effective method for encapsulating natural oils into multifunctional nanofibers that can be used in the pharmaceutical and biomedical fields [22]. Through the controlled regulation of nanofiber degradation, encapsulated bioactive compounds can be released from the nanofibers [22,23]. Within biodegradable polymers, polyvinyl alcohol (PVA) is one of the most widely used and has many good properties that make it suitable for medical applications due to its similarity to natural tissues and its biocompatibility [24,25,26,27,28]. PVA has been used in the medical and textile industries for its non-toxicity, biodegradability, aqueous solubility, chemical resistance, and low environmental impact [29]. Polyvinylpyrrolidone (PVP) is a synthetic polymer characterized by biocompatibility, excellent solubility in most organic solvents, and the ability to interact with hydrophilic materials [30,31]. PVP is widely employed in the pharmaceutical, biomedical, cosmetic, and food industries [32]. Both polymers are approved by the FDA (Food and Drug Administration) for various applications and are estimated as biocompatible polymers in a wide range of areas, including food supplements, embolization, and other in vivo biomedical applications [33,34,35,36,37]. There is an increasing request for preventive and therapeutic strategies but also for cosmetic products containing natural ingredients to counteract the aging process [1].

Plants are a primary source of several bioactive molecules that have been used since ancient times in health promotion. Monoterpenes and sesquiterpenes are secondary metabolites of plants and the main components of essential oils used in folk medicines, pharmaceutical industries, and cosmetics [38]. Natural compounds act as antioxidants to prevent processes that adversely influence skin health, protecting it from aging and exposure to environmental factors [39]. *H. italicum* oil (*HO*) is one of the most widely used essential oils in cosmetics, stimulating the microcirculation of the skin, strongly regenerating it, and helping to reduce the formation of wrinkles [38]. Several studies demonstrated that *HO*, and the notable bioactive compounds contained in the plant, exhibit antioxidant and antibacterial activity [40]. Antioxidants are efficient scavengers of ROS and reducers of oxidative damage, and these compounds have been investigated for therapeutic approaches for many different diseases. The antioxidant activity of *H. italicum* extract was evidenced through its radical scavenging activity, which can restore cellular redox balance [41]. Specifically, experiments performed in vitro and in vivo demonstrated the antioxidant power of H. italicum essential oils against superoxide radicals and lipid peroxidation [42]. Within this context, we synthesized different types of nanofibers and combined them with HO to evaluate their effect in protecting stem cells and the human BJ cell line of foreskin fibroblasts from UV light-induced stress.

## 2. Results

### 2.1. Essential Oil Composition

The GC-MS analysis highlighted 35 distinct compounds, comprising five monoterpenes, nine oxygenated monoterpenes, fourteen sesquiterpenes and five oxygenated sesquiterpenes (Table 1).

### 2.2. Nanofibers Features

**Figure 1:**
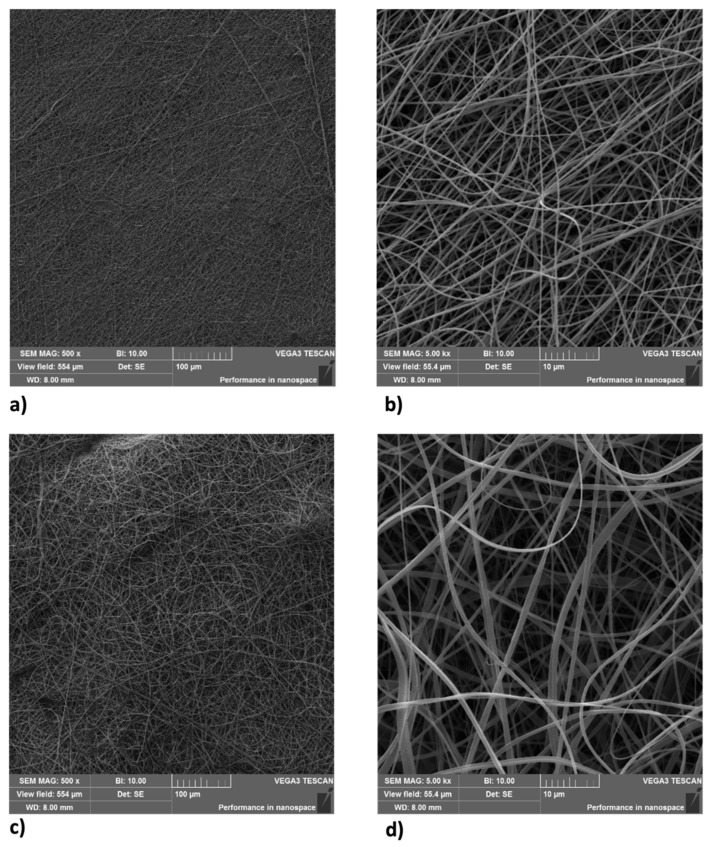
Images acquired with SEM microscope showing PVA 1% (sample 1) and PVP 1% (sample 2) nanofiber structure. Panel (**a**) and panel (**c**) shows nanofiber at 500× magnification. Panel (**b**) and panel (**d**) shows nanofiber at 5000× magnification.

**Figure 2:**
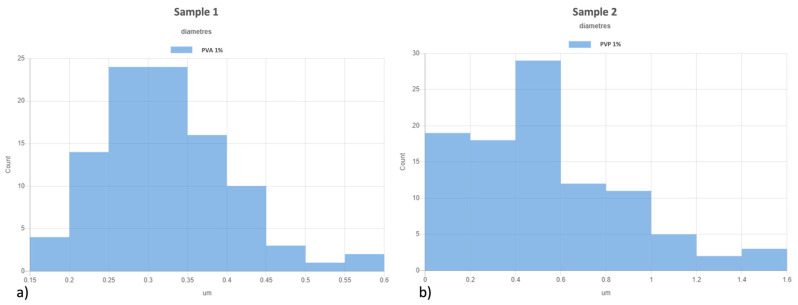
Images show histograms of fibers’ diameters. Panel (**a**) sample 1 (PVA1%) and panel (**b**) sample 2 (PVP1%).

All samples were studied with a scanning electron microscope (SEM) (Device Vega 3 from Tescan, Brno, CZ Czech Republic) after spinning due to sputtering tiny samples using a thin film of gold (Quorum Q150R S, Laughton, UK). Figure 1a–d shows the morphology of fibers. Figure 2 shows histograms of diameters of each sample. Electrospinning is a self-organized procedure, so the orientation of fibers is random. The fibers of sample 1 (Figure 1a) are uniform in diameter with an average of 365.5 nm with a standard deviation of 95 nm. Sample 2 (Figure 1d) has more ribbon-like fibers with a higher average of 709 nm, sd 479.5 nm. The layer density of all samples was about 10 gsm.

### 2.3. PVA and PVP Pretreatment Maintain Cell Viability under UV Stress

**Figure 3:**
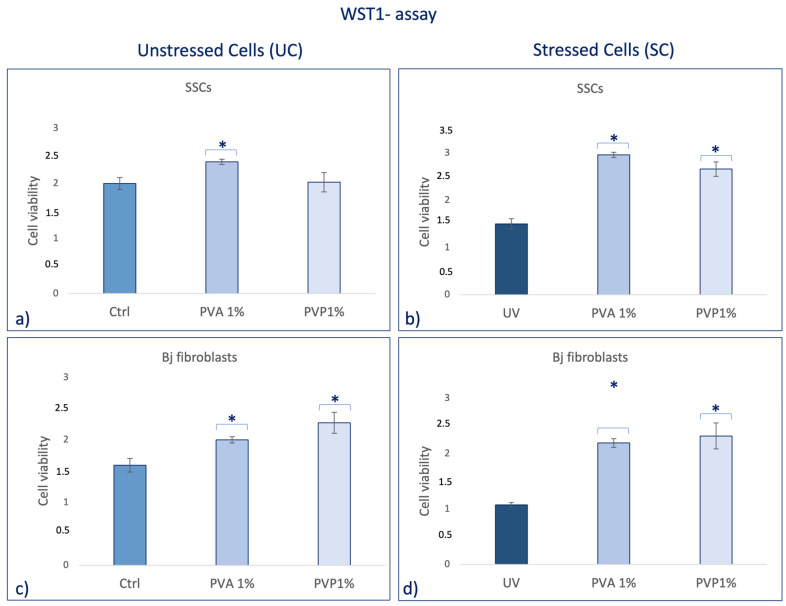
WST1 assay in SSCs and BJ Fibroblasts after 2 h of treatment with PVA 1% and PVP 1% nanofibers. Panel (**a**,**c**) show cells cultured for 2 h with sample 1 (PVA 1%) and sample 2 (PVP1%) and not exposed to UV (UC). Panel (**b**,**d**) show pretreated cells for 2 h with sample 1 (PVA1%) and sample 2 (PVP 1%) and then exposed to UV (SC). Error bars represent standard deviation. * *p* value ≤ 0.05.

Human skin stem cells (SSCs) cultured with PVA1% or PVP1% for 2 h show an increase in viability (Figure 3a) as compared to the untreated control (Ctrl), being statistically significant only for PVA1%-treated cells. Pretreatment of SSCs (Figure 3b) with PVA1% and PVP1% before UV stress, induced a significant increase in cell viability, as compared to the positive control (UV). On the other hand, when SSCs were treated with PVP1% (Figure 3a), they showed only a faint proliferation rate increase compared to the untreated control (Ctrl). BJ fibroblasts cultured in the presence of both PVA1% and PVP1% show a significant increase in viability compared to the untreated control (Figure 3c). Interestingly, the pre-treatment of BJ fibroblasts with both PVA and PVP, encapsulated with 1% *HO* before UV exposure, was able to induce a significant increase in cell viability, as compared to the control stressed cells (Figure 3d).

### 2.4. PVA1% and PVP1% Protect Cells from UV-Induced Senescence

**Figure 4:**
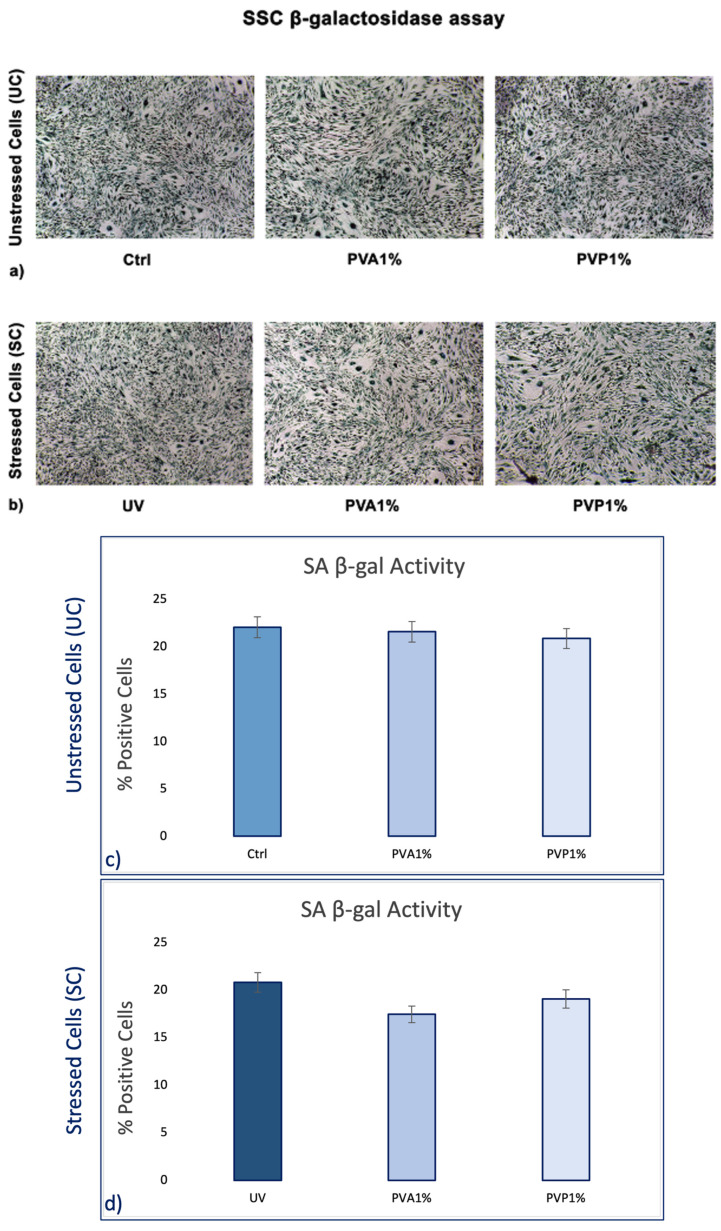
Senescence-associated β-galactosidase activity was evaluated in SSCs after 2 h of culturing with PVA and PVP, encapsulated with 1% *HO* before and after exposure to UV light. Scale bar = 100 μm (panels **a** and **b**). The number of positive (blue) cells for each condition was calculated as the number of positive cells divided by the total number of cells counted using an image software analysis (ImageJ, Version 2.14.0). Data are expressed as mean ± SD referring to the control (panels **c** and **d**).

β-galactosidase colorimetric assay shows that the number of blue positive cells was reduced when they were pre-treated with nanofibers, for both PVA and PVP, encapsulated with 1% *HO* before exposure to UV light, as compared to the positive controls (UV) (Figure 4a). No differences were observed in the absence of UV exposure, although it can be observed that the number of positive cells is slightly lower than in the untreated controls (UC) for both treatments (PVA and PVP) (Figure 4b).

### 2.5. PVA1% and PVP1% Pretreatment Promote a Molecular Program of Youngness

**Figure 5:**
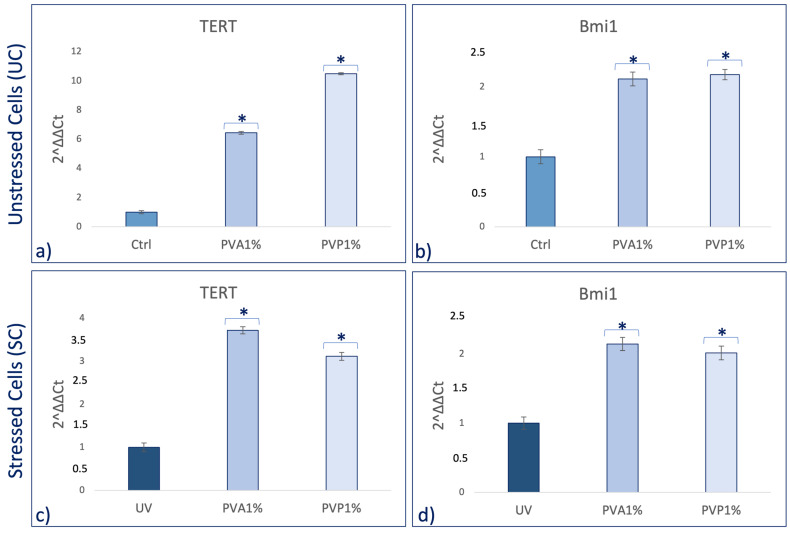
Effect of PVA1% and PVP1% treatment on the expression of TERT (**a**,**c**) and Bmi1 (**b**,**d**) in SSCs. SSCs were exposed for 2 h with PVA1% and PVP1% and then stressed (**c**,**d**) or not (**a**,**b**) with UV. The amount of mRNA was normalized to GAPDH and was plotted as fold change (2^−∆∆CT^) as compared to controls. * *p* value ≤ 0.05.

**Figure 6:**
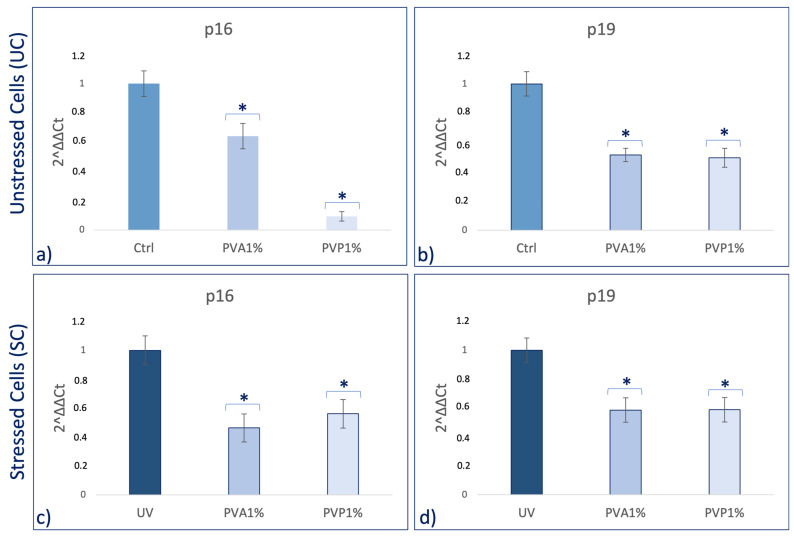
Effect of PVA1% and PVP1% treatment on the expression of p16 (**a**,**c**), p19 (**b**,**d**) on SSCs. SSCs were exposed for 2 h with PVA1% and PVP1% and then stressed (**c**,**d**) or not (**a**,**b**) by UV. The amount of mRNA was normalized to GAPDH and was plotted as fold change (2^−∆∆CT^) as compared to controls. * *p* value ≤ 0.05.

**Figure 7:**
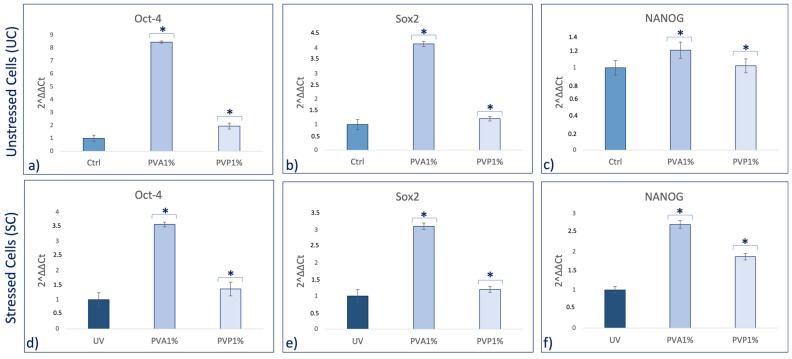
Effect of PVA1% and PVP1% treatment on the expression of Oct-4 (**a**,**d**), Sox2 (**b**,**e**) and NANOG (**c**,**f**) on SSCs. SSCs were exposed for 2 h with PVA1% and PVP1% and then stressed (**d**–**f**) or not (**a**–**c**) by UV. The amount of mRNA was normalized to GAPDH and was plotted as fold change (2^−∆∆CT^) as compared to controls. * *p* value ≤ 0.05.

Real-time qPCR reveals that pretreatment with PVA and PVP, encapsulated with 1% *HO,* before UV exposure was able to prevent the UV-induced downregulation of TERT and Bmi1 gene expression on SSCs (Figure 5c,d). Concomitantly, the same pretreatment inhibited the appearance of the senescence-regulating genes p16 and p19 (Figure 6c,d). Moreover, the expression of the stemness genes Oct-4, Sox2, and NANOG was significantly increased on skin stem cells pretreated with both PVA1% and PVP1% and then exposed to UV (Figure 7d–f).

## 3. Discussion

Skin aging is a biological process closely related to senescence and is increased by exposure to extrinsic factors, such as photodamage [43]. Aging induces cellular damage and changes in the extracellular matrix composition (ECM), also influencing stem cell behavior [44]. Stem cells, supported by fibroblasts, play an important role in the ECM, being directly involved in the aging process of tissues exposed daily to sunlight [45,46,47,48]. Other authors demonstrated the role of UV in the skin aging process. Indeed, solar radiation causes oxidative stress, thus compromising the function of the skin barrier, making it more sensitive [49]. Natural extracts, as helichrysum oil, appropriately delivered by biocompatible devices can improve skin repairing processes after damage. Within this context, in traditional medicine, Helichrysum oil is recognized for its antioxidant and anti-inflammatory properties on the skin. The chemical composition depends on the geographical origin and the plant material being processed [50]. In this study, phytochemical investigations suggest the presence of terpenes comprising monoterpenes, oxygenated monoterpenes, sesquiterpenes, and oxygenated sesquiterpenes (Table 1). Terpenes present a wide range of biological properties to be considered for their use in modern medicine. They are recognized as stimulators of skin penetration, agents involved in the prevention and therapy of different inflammatory diseases, also influencing skin permeability [50]. The main outstanding characteristics of electrospun nanofibers that meet the objectives and features of this study are versatility, flexibility, as well as nano-biomimetic features, and similarity to extracellular matrix components. They can be functionalized on both the surface and the core, becoming efficient and affordable tools for embedding a wide variety of bioactive players. PVA-based nanofiber membranes are a medical device that may be used to develop novel medicinal applications [51]. PVA is a biological polymer known for its use for pharmaceutical purposes in tablets and hydrogels containing bioactive drugs in controlled-release systems [52,53]. PVP was one of the first synthetic polymers to be tested as an artificial cartilage due to its adaptability, water solubility, and very low cytotoxicity [54]. It has recently been described for its use in transdermal drug delivery applications [55]. This polymer has been approved by the US Food and Drug Administration as a safe polymer for pharmaceutical and biomedical experiments [56]. Electrospinning is a nanotechnology fiber-forming method that produces these submicrometer fibers from polymer solutions and melts [57]. Fibers with diameters in the range of a few micrometers to tens of nanometers can be produced by modulating the properties of the polymer solution (e.g., concentration, viscosity, and molecular weight of the polymer), the electrospinning setup (e.g., spinning electrode, collector, flow rate, collector distance, and applied voltage), as well as environmental properties (e.g., humidity and temperature). Therefore, the electrospinning technique allows for the creation of membranes with nanoscale topography, variable porosity, and huge surface-area-to-volume ratios [58]. Functionalized nanofibers created through “coaxial electrospinning,” a sophisticated method of producing the next-generation nanofibers with a specifically organized core-shell structure, are an appealing strategy for delivering susceptible biomolecules because the produced core-shell fibers have a high potential for protein preservation during the electrospinning process [59]. Nanofibers are an interesting material themselves, but their primary potential in medicine appears to be kept in their future functionalization, i.e., the fabrication of so-called smart nanofibers. This is obtained by modifying the nanofiber surface and/or core to induce new characteristics. There are several methods for modifying nanofiber surfaces, among which the most common is a physical modification by cold plasma, followed by a specific chemical intervention resulting in the attachment of a specific molecule (e.g., miRNA) [60,61]. The surface and core of nanofibers can be used to immobilize a variety of bioactive molecules like RNA, protein molecules, antimicrobial agents, and other medicines. Recent advances in manufacturing protein-compatible nanofibers have broadly opened the door for additive manufacturing (AM) techniques, which are regarded as a revolutionary tool for creating customized microarchitectures for smart scaffolds and active drug delivery systems [62]. Nanofiber membranes are known for their versatility in incorporating drugs and releasing them over a long period of time [63]. In the present study, we applied nanotechnologies such as PVA and PVP nanofibers combined with Helichrysum Oil to allow a delivered pretreatment on UV-stressed cells. The combined nanofibers were characterized by scanning electron microscopy for morphology (Figure 1 and Figure 2). The activity of the nanofibers was tested in vitro in SSCs and BJ fibroblasts. In both cell lines, we showed that these functionalized nanofibers were able to increase viability, counteracting aging triggered by UV stress (Figure 3b,d). Moreover, the β-galactosidase assay, performed on stem cells, highlights the protective effect of PVA1% nanofibers on aging induced by UV irradiation (Figure 4b). p16 and p19 genes are induced to stop the cell cycle when cells are damaged and after prolonged cell division [40]. Bmi1 represses genes involved in senescence and, at the same time, probably induces telomerase activity to prevent telomere shortening [64]. p16 and p19 are expressed when Bmi1 is transcriptionally downregulated, as cells that undergo replicative senescence [65]. At the same time, Bmi1 modulates stemness genes, as Oct 4 and Sox2(66). Oct-4, Sox-2, and NANOG influence stem cells’ plasticity and regenerative capability, especially during the senescence process, when their activity gradually decreases [66,67]. The molecular senescence program activated after UV exposure was counteracted by pretreatment with PVA1% and PVP1% in stressed cells (Figure 5c,d, Figure 6c,d and Figure 7d–f). In our experiments, we could detect a downregulation of both p16 and p19 genes in stem cells pretreated with both PVA1% and PVP1% and then exposed to UV stress, as compared to control un-pretreated, stressed cells (UV). Both nanofibers, especially PVA1%, show a protective effect, saving cells from the cell cycle exit and thus from the appearance of a senescent phenotype (Figure 6). Pretreatment with PVA1% and PVP1% before UV exposure increased the expression of Bmi1, Oct-4, Sox2, and NANOG while at the same time, downregulating p16 and p19 (Figure 6c,d and Figure 7d–f). Moreover, TERT, the promoter gene regulating telomerase activity, was positively modulated by PVA1% and PVP1% nanofibers, both under stressed and unstressed conditions (Figure 5a,c) [68]. We demonstrated that PVA and PVP nanofibers combined with helichrysum oil can counteract photoaging induced by UV, accelerating rejuvenation processes, by acting on the molecular pathway of pluripotency and cellular senescence. This finding may pave the way to novel strategies aimed at preventing, counteracting, and potentially reversing both intrinsic and extrinsic skin aging. Moreover, although elderly subjects retain the capability of healing wounds, they exhibit a slower healing process, affecting all phases of wound healing, as compared with young individuals. The present observations may therefore provide novel perspectives for turning the slow process of elderly wound healing into a rejuvenated, faster dynamic.

## 4. Materials and Methods

### 4.1. Extraction of Helichrysum Italicum Essential Oil

The essential oil samples were obtained from young stems by hydrodistillation in a Clevenger-type apparatus for 1 h with 500 mL of distilled water, following an established protocol [69]. Subsequent GC-MS analysis was carried out using a Hewlett Packard 5890 GC-MS system operating in EI mode at 70 eV and equipped with either (1) an HP-InnoWax capillary column (30 m × 0.25 mm, film thickness 0.17 mm), over a temperature gradient of 4 °C per minute, starting at 60 °C for three minutes and ending at 210 °C for 15 min; or (2) a HP-5 capillary column (30 m × 0.25 mm, film thickness 0.25 mm) over a temperature gradient of 4 °C per minute, starting at 60 °C for three minutes and ending at 300 °C for 15 min. The injection and transfer line temperatures were 220 °C and 280 °C, respectively. Helium was used as the carrier gas at 1 mL per minute flow rate and a 1:10 split ratio.

### 4.2. Identification of Components of HO

The identification of components was achieved by comparing the GC retention index (RI) on the apolar and polar columns with those of authentic samples of various essential oils and by matching the MS fragmentation patterns and retention index with stored Wiley 7 mass computer library, NIST (National Institute of Standards and Technology) or data in the literature [70]. A hydrocarbon mixture of alkanes (C9-C22) was analyzed separately under the same chromatographic condition to calculate the RIs using a generalized equation [71]. The following standards were included: linalool (Purity ≥ 95%, Fluka), 1,8-cineole (99% purity, Aldrich), nerol (Purity ≥ 90%, Fluka), geraniol (Purity ≥ 96%, Fluka). C9-C22 alkane standards (purity 98–99%) were purchased from Aldrich.

### 4.3. PVA and PVP Electrospun Nanofibers Production and Combination with HO

Nanofibers were fabricated by an electrospinning method using a Nanospider 1S500U machine (Elmarco s.r.o. Liberec, Czech Republic). Electrospinning was performed using a needleless wire electrode to collecting substrate from spunbond textile (PF Nonwovens, Prague, Czech Republic). Spinning parameters are shown in Table 2. The solutions for the electrospinning process containing 10% *w*/*w* of PVA (Mowiol 5–88 + 40–88 in 20% *v*/*v* ethanol with deionized water) or 12% of PVP (Sigma PVP360) in ethanol (*w*/*v*) 1 h before spinning was added to 1 g of polymer 1% of oil described above (about 8 gtt-10%/0.01 g 2 gtt 1%) >and mixed at 600 rpm. Oil was not in contact with (potentially) harmful organic solvents. 

### 4.4. Cell Isolation and Culturing

SSCs were obtained from biopsies of adult male and female patients after ethics committee approval (Ethical Clearance N. 0021565/2018, 22/03/2018-Commissione Etica CNR). They were isolated and cultured as previously described [72]. BJ fibroblasts (#08-0027, Stemgent, Cambridge, MA, USA) were isolated from human normal neonatal foreskin and cultured in a Dulbecco’s modified Eagle’s Medium (DMEM) low-glucose medium (Life Technologies, Carlsbad, CA, USA), supplemented with 10% fetal bovine serum (FBS Life Technologies, Carlsbad, CA, USA), 2 mM l-glutamine (Euroclone, Milano, Italy) and 1% of penicillin/streptomycin (Euroclone, Milano, Italy).

### 4.5. Experimental Design

BJ fibroblasts and SSCs were cultured under two experimental conditions. A group of cells was maintained in a basic growing medium, without UV light exposure (Ctrl). A group of cells was cultured in basic growing medium and then exposed to UV light (UV). A group of cells was pre-treated with encapsulated nanofibers without exposure to UV light (Unstressed Cells, UC), and the last group of cells was pre-treated with encapsulated nanofibers and then exposed to UV light (Stressed Cells, SC). Pre-treatment was performed using electrospun nanofibers PVA and PVP functionalized with 1% oil. Cells were exposed directly to UV light for 3 min at a 10 cm distance from the lamp to induce stress. All the conditions used are summarized in Table 3.

### 4.6. Evaluation Cell Viability

The water-soluble tetrazolium salt assay (WST-1) (Sigma-Aldrich, Saint Louis, MO, USA) was performed to evaluate the metabolic activity of BJ Fibroblasts and SSCs. Cells were seeded at a concentration of 12.000 cells/well in a 96-well plate and treated with sample 1 (PVA1%) and sample 2 (PVP1%) for 2 h. After culturing in the above-described conditions, the WST-1assay (Sigma-Aldrich) was performed in cells that were exposed and not exposed to UV light. Cell viability was detected by a plate reader (OD 450 nm) and expressed in OD units as compared to untreated cells (Ctrl). Data were expressed as mean ± SD, referring to the control.

### 4.7. Senescence-Associated β-Galactosidase Staining

The ability of PVA1% and PVP1% to prevent aging was evaluated by β-Galactosidase assay. Cells pretreated with the nanofibers for 2 h and then exposed (SC) or not exposed (UC) to UV were stained according to the manufacturer protocol instructions, using the senescence-associated (SA) β-Galactosidase Staining Kit (Cell Signaling Technology, Euroclone, Milan Italy). Qualitative detection of SA-β-Galactosidase activity, visible thanks to the blue coloration, was detected under an inverted microscope (Magnification 4× bright field). Control and UV cells were used as negative and positive controls for senescence staining. Quantitative detection was performed using an image software analysis (ImageJ, version 1.8.0, National Institutes of Health, Bethesda, MD, USA).

### 4.8. Real Time-qPCR

Gene expression levels were detected by Real Time-qPCR in SSCs after 2 h of treatment with sample 1 (PVA 1%) and sample 2 (PVP 1%) before exposure to UV light. Total mRNA was isolated using RNeasy Mini Kit (Qiagen, 40724 Hilden, Germany) according to the manufacturer’s protocol. The quantity and purity of RNA were measured by OD 260/280 nm using a Nanodrop (Thermo Fisher Scientific, Waltham, MA, USA). Then, 2.5 ng of RNA from each sample in triplicate was reverse-transcribed and amplified by a Luna^®^ Universal One-Step RT-qPCR Kit (New England Biolabs, 240 County Road Ipswich, MA, USA) via the Thermal Cycler (Bio-Rad, Hercules, CA, USA). The RT-qPCR analysis was performed for the stemness markers Oct-4, Sox2 and NANOG, for the cell cycle-related genes, p16 and p19, and for Bmi1 and TERT. All the primers were previously described [73,74]. Resulting Ct (Threshold Cycle) values normalization was performed on the housekeeping HPRT1 (Hypoxanthine Phosphoribosyltransferase 1) and mRNA levels were expressed as fold of change (2^−∆∆Ct^) of expression levels of positive (UV) and untreated controls (Ctrl), as previously described [75,76].

### 4.9. Statistical Analyses

The experiments were performed two times with three technical replicates for each treatment. Two-way analysis of variance ANOVA tests with Tukey’s correction and the Wilcoxon signed-rank test were used, assuming a *p* value < 0.05 as statistically significant. We considered * *p* < 0.05.

## 5. Conclusions

A combination of nanocomposites and natural compounds was shown to improve the specific functionalities of synthesized polymers. Indeed, *HO* combined with PVA and PVP nanofibers, gives them properties such as controlled release, biocompatibility, and biodegradability. Indeed, our results showed that PVA1% and PVP1% nanofibers are most effective especially when cells are exposed to stress. In this paper, we show that nanofibers encapsulated with *Helichrysum Italicum* oil (*HO*) could be successfully applied to prevent the aging process, without adverse effects on skin. The nanofibers, already recognized for their biomedical characteristics, showed an immediate degradation compatible with cell populations and produced a quick stress response after only two hours of treatment. New approaches such as an encapsulation of traditionally used, well-known active molecules into suitable biocompatible materials using electrospinning can provide a new perspective for long-established areas and become a gamechanger for widespread well-being and therapeutic applications. In conclusion, these nanofibers, which are biocompatible and safe for human health, could potentially be used as a medical device, making them excellent candidates for topical application.

## Figures and Tables

**Table 1:** Results of GC-MS analysis show the groups of compounds present in *HO*.

Monoterpens	α-pinene, α-fenchene, β-pinene, Limonene, γ-terpinene
Oxygenate Monoterpens	Linalool 1,8-Cineolo, Neryl propionate, Neryl isobutanoato, Neryl isovalerato, Nerol, Nerolidol, Nerol oxide, Neryl acetato, Geraniol
Sesquiterpens	Italicene, Iso-Italicene, Caryofillene, γ-Curcumene, Ar-Curcumene, cis-β-Guaiene, cis-α-Guaiene, trans-β-Guaiene, γ-Cadinene, δ-Cadinene, α-Cadinene, α-cis-Bergamotene, α-trans-Bergamotene, Alloaromandrene
Oxigenate Sesquiterpens	τ-Cadinolo, Guaiol, β-Eudesmol, α-Eudesmol, Eudesm-5-en-11-ol

**Table 2:** Spinning parameters of sample 1 (PVA1%) and sample 2 (PVP 1%).

Spinning Parameters	Sample 1 PVA 1%	Sample 2 PVP 1%
Voltage (collector electrode)	20 kV	20 kV
Voltage (spinning electrode)	30 kV	25 kV
Distance between electrodes	18 cm	18 cm
Collecting substrate speed (mm/min)	5	5
Temperature	21 °C	21 °C
Humidity	30% RH	30% RH

**Table 3:** Cell culturing conditions.

Experimental Conditions	
(Ctrl) Untreated Control	- T - UV
(UV) Positive control	- T + UV
(UC) Unstressed Cells	+ T - UV
(SC) Stressed Cells	+ T + UV

(- without, + with); T = treatment with HO combined with nanofibers; UV = exposition to UV light.

## Data Availability

The data of the current study are available inside of the manuscript.
